# Mixed histiocytosis of Langerhans cell with Rosai-Dorfman disease

**DOI:** 10.1016/j.jdcr.2025.02.013

**Published:** 2025-03-08

**Authors:** Avery DeLong, David Othman

**Affiliations:** a University of Toledo College of Medicine and Life Sciences, Toledo, Ohio; b Department of Dermatology, Cook County Health, Chicago, Illinois

**Keywords:** BRAFV600E, dabrafenib, histiocytosis, intracranial lesions, Langerhans cell, pruritic inguinal rash, Rosai-Dorfman

## Introduction

Histiocytoses are a group of rare proliferative disorders that share a common CD34+ progenitor cell in the bone marrow. They can be classified as either Langerhans cell histiocytosis (LCH), non-Langerhans cell histiocytosis, or malignant histiocytic disorders. Mixed histiocytosis is an emerging group of syndromes defined by the overlap of Langerhans cell histiocytosis and another histiocytic disorder of a different type. Despite being rare, it may account for up to a fifth of systemic histiocytosis patients.^1^ LCH is the most common histiocytosis in the United States and presents in a few 100 patients every year. Whereas Rosai-Dorfman disease (RDD), a type of non-Langerhan histiocytosis is much rarer with only 1000 cases reported worldwide as of 2020.^2^^,^^3^ The co-occurrence of both LCH and RDD is quite remarkable, and we present a case of this type of mixed histiocytosis to contribute to the scarce existing literature.

## Case report

**Fig 1:**
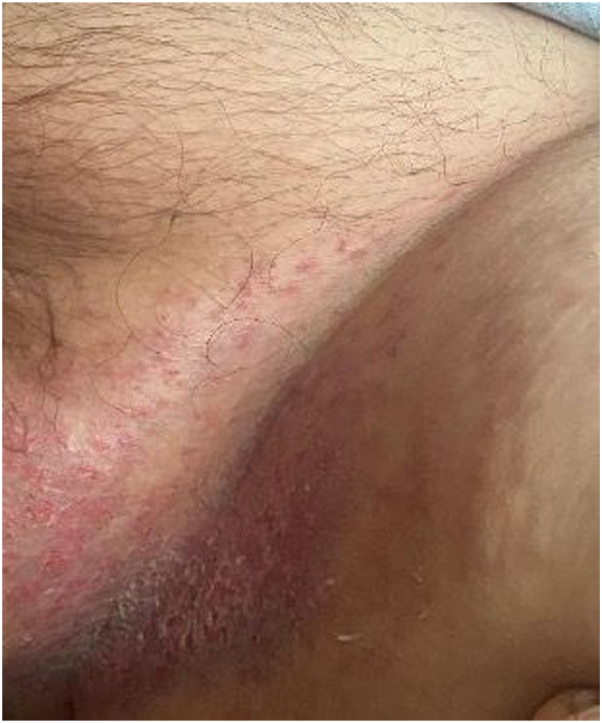
In the inguinal folds there were symmetric, well-circumscribed erythematous, eroded plaques with peripheral scale.

A 58-year-old man with a 6-year history of diabetes insipidus and a 3-month history of panhypopituitarism presented with blurry vision, headaches, dizziness, and diaphoresis. An MRI of the brain revealed intracranial lesions, and the patient was admitted for further work-up. Dermatology was consulted for a pruritic inguinal rash that had been present for the last 2 years and had been unresponsive to topical antifungals (Fig 1). The patient’s past medical history was notable for type 2 diabetes, hypertension, and a relatively new diagnosis of diabetes insipidus and panhypopituitarism. His lab abnormalities were relatively minimal.

**Fig 2:**
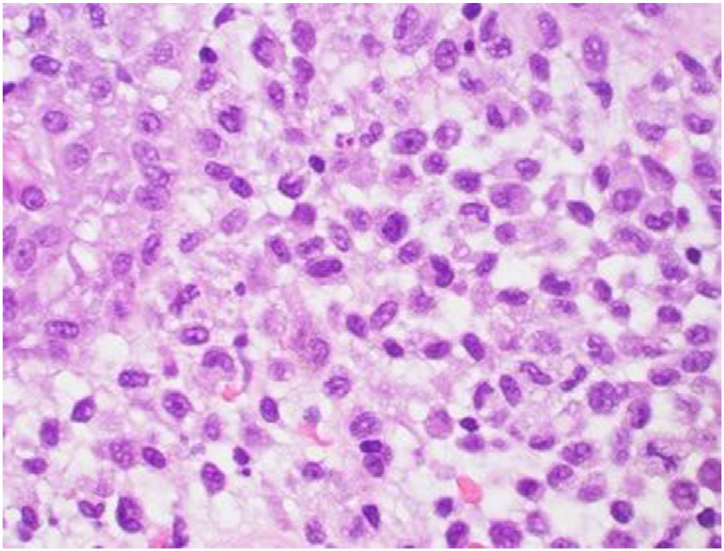
Higher 40× power revealed cells with reniform nuclei and pink cytoplasm.

With a punch biopsy of these inguinal folds, lower power demonstrated hyperplasia of the epidermis, and a dense infiltrate in the superficial dermis. The cells seen in Fig 2 were CD1a and langerin positive, consistent with LCH. Further workup with imaging included a CT scan of the head and an MRI of the brain, which both revealed 3 intracranial masses in the frontal lobe, suprasellar cistern, and medial aspect of the right inferior lobe. A brain biopsy of one of the lesions was performed and showed a mixed infiltrate of histiocytes, plasma cells, and lymphocytes.

**Fig 3:**
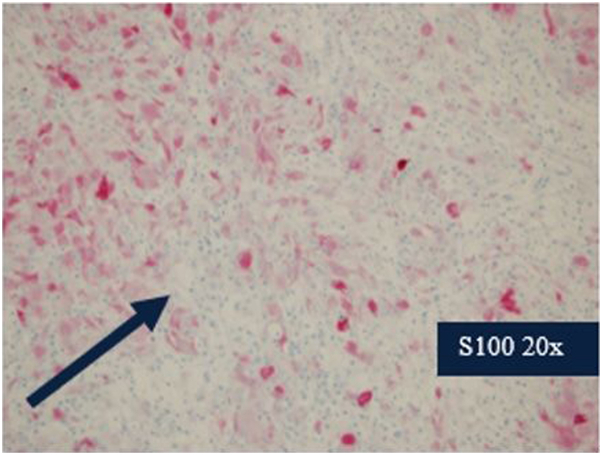
Here, we see 20× magnification of the brain biopsy stained with s100 highlighting the histiocytes.

In Fig 3, there are lymphocytes present within the cytoplasm of histiocytes which is known as emperipolesis. The final diagnosis was Langerhans cell histiocytosis with RDD, a type of mixed histiocytosis. After genetic testing revealed a positive BRAF V600E mutation in both the skin and brain biopsies, hematology started the patient on dabrafenib, a BRAF inhibitor. After several months of therapy, the patient’s cutaneous lesions nearly resolved, intracranial lesions decreased in size, and his overall cognition improved. He continues to follow up with other medical specialties and receives multidisciplinary care.

## Discussion

LCH is the most common histiocytic disorder and is driven by mutations in the mitogen-activated protein kinase pathway. It typically presents between the ages 1 and 3, although in rare cases it can present later in life. LCH can range from asymptomatic to mild, or a single-organ disease to severe, progressive multisystem disease. Classic cutaneous findings consist of erythematous to brown papules and plaques on the scalp and skin folds. When there is systemic involvement, LCH commonly affects the bone, liver, lung, and pituitary.^4^ Our patient presented with both cutaneous findings as well as pituitary involvement, specifically diabetes insipidus, and panhypopituitarism.

In addition to his diagnosis of LCH, our patient was found to have a concomitant non-Langerhans cell histiocytosis, RDD. In the systemic form of RDD, as seen in our patient with brain involvement, only 10% of cases have cutaneous findings. Classic cutaneous findings when present include red to brown papules, nodules, or plaques most commonly located on the eyelids or malar areas. Noncutaneous findings of RDD classically consist of significant lymphadenopathy, while 40% of patients can have extra-nodal involvement, affecting the soft tissue, bone, ocular region, salivary glands, upper respiratory tract, or central nervous system.^4^ Our patient presented with intracranial lesions without concomitant lymphadenopathy, which is a less common presentation.^5^

**Fig 4:**
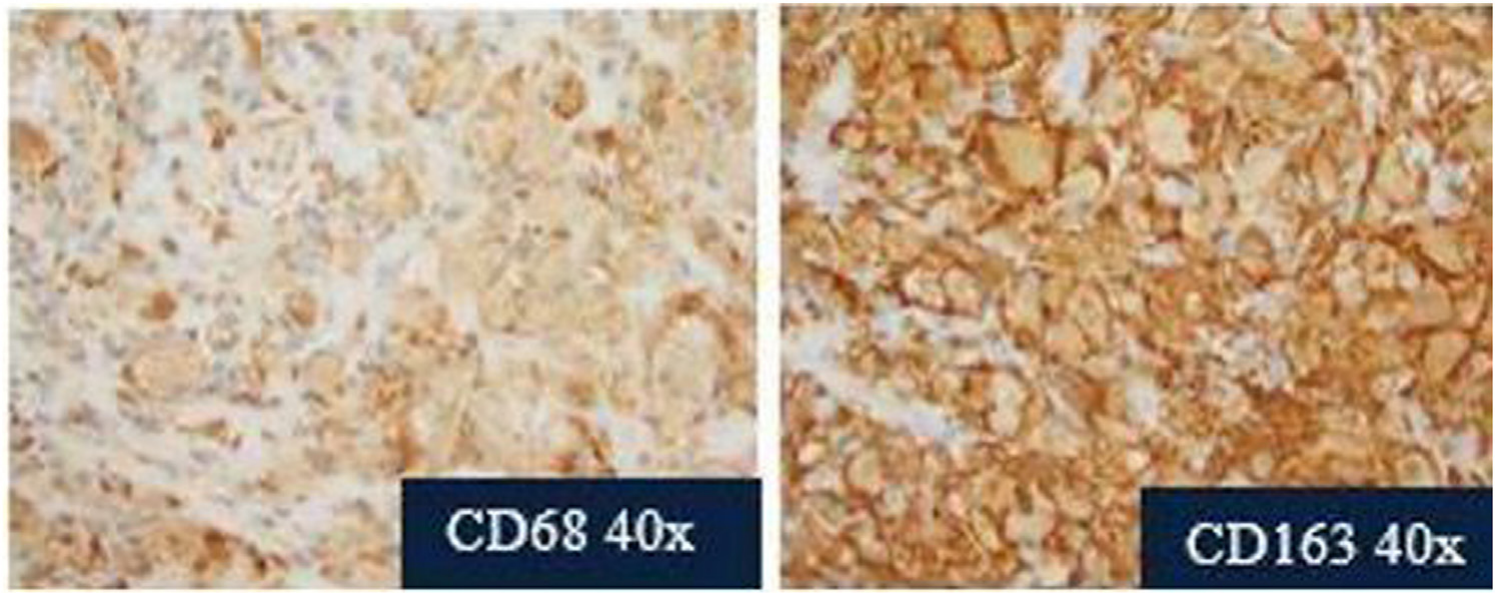
Further stains demonstrated findings of cells that were positive for CD68 and CD163 while being negative for Langerin and CD1a. These findings were consistent with RDD. *RDD*, Rosai-Dorfman disease.

LCH and RDD can be differentiated by histology and immunohistochemistry. LCH classically stains positive for S100, CD1a, Langerin (CD207), and CD68. On the other hand, RDD stains positive for S100, CD68, and CD163, and is negative for CD1a and Langerin.^3^^,^^5^ The presence of reniform nuclei in LCH and emperipolesis in RDD can aid in the diagnosis of these entities (Fig 4).^3^

A mixed histiocytosis, such as concomitant LCH and RDD, as was seen in our patient, is rare.^1^^,^^3^ Mixed histiocytosis, defined as the overlap of LCH with another type of histiocytosis, has been cited in a recent review article of 105 cases.^1^ It was noted that patients with a mixed histiocytosis were more likely to have a *BRAF-V600E* mutation, as was seen in our patient.^1^^,^^5^ Genetic testing can be very helpful in guiding targeted therapy in such cases. BRAF inhibitors, such as dabrafenib^6^^,^^7^ or vemurafenib,^8^ have been used as therapeutic agents. Given the paucity of overall cases, there are limited data on the efficacy of BRAF inhibitors in the treatment of mixed histiocytoses. BRAF inhibitors have shown results ranging from rapid and clinical remission^7^ to minimal clinical improvement in 57% of histiocytosis patients.^8^ In our case, the patient had a steady response to dabrafenib over the past couple of months, with both clinical and radiographic improvement. However, it is also important to note that a *BRAF-V600E* mutation is associated with an increased risk for relapse.

Diagnosing a histiocytosis can be difficult, given its varied clinical presentation. For dermatologists, it is prudent to keep this entity in our differential diagnosis for inguinal rashes, especially when atypical or unresponsive to traditional therapies. Additionally, the presence of systemic findings such as diabetes insipidus should further lower the threshold for suspecting a histiocytosis and thus prevent a delay in diagnosis.

In conclusion, a mixed histiocytosis with coexisting LCH and noncutaneous non-LCH in a patient is uncommon. Often the clues to diagnosis are systemic findings; however, a skin biopsy can be diagnostic in these challenging cases. Additionally, in patients with multiple histiocytosis, it is important to perform genetic testing to evaluate for a *BRAF-V600E* mutation to ensure that patients receive the appropriate targeted therapies.

## Conflicts of interest

None disclosed.
